# Phenolic composition, antioxidant capacity, enzyme inhibition, and aroma composition of lyophilized olive leaves of cv. Gemlik and Kilis Yağlık

**DOI:** 10.1007/s13197-026-06662-3

**Published:** 2026-04-15

**Authors:** Gulcan Koyuncu, Tugba Kilic

**Affiliations:** Department of Food Processing, Technical Sciences Vocational School, Kilis 7 Aralik University, 79000 Kilis, Turkey

**Keywords:** Olive leaves, LC-HRMS, α-glucosidase, α-amylase, SPME

## Abstract

In this study, lyophilized leaves from olive varieties grown in Turkey were extracted using three different solventswith ultrasonication. Antioxidant capacity, total phenol, phenolic compounds, and enzyme inhibition of the extracts were determined. By LC-HRMS, 27 compounds among 84 different phenolic standards were determined in olive leaves, and approximately 35% of them were oleuropein. Total phenol content varied depending on solvents and variety, and antioxidant capacity varied accordingly. Kilis Yağlık ethanol and Gemlik methanol extracts showed the highest inhibition of α-glucosidase and α-amylase, respectively. The aroma compounds of the leaves were extracted by solid-phase microextraction (SPME) and identified by GC-MS. A total of 52 volatile compounds belonging to seven different classes were identified in olive leaves. Considering the obtained bioactive components and properties, the antidiabetic effect of olive leaves and the volatile compounds in their composition are noteworthy. Overall, this study highlights the valorization of olive leaves as an agro-industrial bio-waste within a circular economy framework, demonstrating how BioE3-driven processing strategies (bioeconomy, bioenergy, and bio-based products) can enable sustainable value addition and resource efficiency.

**Graphical abstract:**

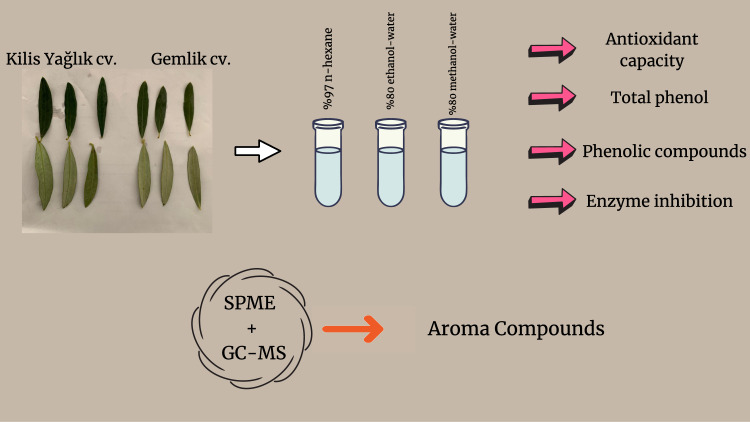

## Introduction

The evaluation of plant-based wastes, both due to their environmental impacts and in other products, attracts great attention in terms of zero waste policies and sustainable food production systems (Zandona et al. 2024). Leaves, which constitute 10% of the olive harvest weight, are obtained as waste during the processing of olives and pruning of trees (Hassan et al. 2024). Olive leaves have recently been evaluated especially in areas such as food and nutraceuticals. Olive leaves can be transformed into value-added products, especially after being processed with green technologies (Alvares et al. 2024). There are studies on adding olive leaves to foods to improve various properties of foods such as bioactivity, aroma, and shelf life. Generally, olive leaves are used in products such as milk and dairy products (milk, yogurt, kefir), meat and its products, cereal products, fruits, vegetable products, beverages, sauces, and edible oils (Salik and Cakmakci 2021). It is known that olive leaves generally do not have toxic effects even in high doses.

Olive leaves contain many bioactive compounds with antioxidant, antimicrobial, and hypoglycemic properties (Hassan et al. 2024). These bioactive compounds are mainly secoiridoids, phenolic acids, and flavonoids (Zhang et al. 2022). It has been reported that oleuropein, an important bioactive component of olive leaf, affects many diseases, and the improvement it provides on insulin resistance is due to its effect on pancreatic cells (Hassen et al. 2015). Oleuropein is a heterozygous ester of β-glucose and 3,4-dihydroxyphenyl ethanol or hydroxy tyrosol (Hassan et al. 2024).

The use of different forms of plants as flavorings in many sectors such as food and medicine has recently attracted attention. Although there are many studies on the flavor compositions of olive and olive oil, there are limited studies on the volatiles of olive leaves (Brahmi et al. 2012). Olive leaves contain volatile compounds belonging to chemical classes such as ketones, aldehydes, alcohols, and terpenes (Paskovic et al. 2019). These volatiles are formed through various pathways, mainly the lipoxygenase pathway, which converts linoleic and linolenic fatty acids into C6 aldehydes, alcohols, and their esters (Ben-Abdeljelil et al. 2017). Differences in the aroma composition of leaves are influenced by many factors such as season, olive variety, condition of leaves, climate, and geographical conditions (Brahmi et al. 2012).

This study aimed to determine the bioactive compounds and properties of the extracts obtained by ultrasonically assisted extraction method with different solvents (97.0% *n-*hexane, 80% ethanol-water, and 80% methanol-water) of Kilis Yağlık and Gemlik olive leaves, which are widely grown varieties in Turkey. The bioactive components (total phenol using the spectrophotometric method and quantitative phenolic component by LC-HRMS and bioactive properties (antioxidan capacity and enzyme inhibition) of the extracts were determined. The aroma compounds of the leaves were separated by SPME and identified by GC-MS.

## Materıals and methods

### Materials

Olive leaves were collected in January 2023 from cv. Kilis Yağlık and cv. Gemlik olive trees are grown in the Kocabeyli Village of Kilis province in southern Turkey. The leaves were washed with water, air-dried, freeze-dried in a lyophilizer (-50 °C, 0.05 mbar), and stored at -25 °C.

### Extraction of leaves

Each of the two leaf varieties was extracted using an ultrasound-assisted extraction method. 200 mL of solvent (HPLC grade 97.0% *n-*hexane, 80% ethanol-water, 80% methanol-water, v/v) was added to the dried powdered leaves (15 g), homogenized in a blender for 2 min and kept in an ultrasonic bath (Bandelin Sonorex Super RK, Germany) for 45 min at 30 °C. The mixture was centrifuged (Kunota 7780 Japane) at 3500 rpm for 10 min at 20 °C, the supernatant was separated and 30 mL of solvent was added to the solid and the extraction was repeated. After the supernatants were centrifuged, the solvent was evaporated in a rotary evaporator and stored at -25 °C. After extraction, the remaining leaf residues were collected and managed in accordance with laboratory waste disposal regulations, minimizing environmental impact.

### Total phenol content and antioxidant capacity

The total phenol content of the extracts was determined by the Folin Ciocalteu colorimetric method as described by Singleton et al. (1999). The total phenol content of the extracts was given as gallic acid equivalent on a dry weight (DW) basis (mg GAE/g DW). Antioxidant capacity was determined using the DPPH method according to Thaipong et al. (2006). The radical reducing capacity of the extracts was expressed as Trolox equivalent on a dry weight basis (µmol TE/g DW).

### Quantitative analysis of extracts by LC-MS/MS

Phenolic compounds of the extracts were determined using LC-HRMS (Thermo Fisher Scientific) equipped with an Exactive Plus Orbitrap high-resolution MS system. In the system, DIONEX UltiMate 3000 RS autosampler, DIONEX UltiMate 3000 RS pump, DIONEX UltiMate 3000 RS column oven, and ESI interface were used. The Orbitrap-MS instrument was calibrated with positive (Pierce™ LTQ Velos ESI Positive Ion Calibration Solution) and negative calibration solutions (Pierce™ Negative Ion Calibration Solution) using an automatic syringe injector (Thermo Fisher Scientific, USA). The instrument was operated with TraceFinder 3.2 (Thermo Scientific) and data were recorded with Xcalibur software (2.1.0.1140). MerckPurosper^®^ STAR RP-18 endcappedHibar^®^ HR (100 mm × 2.1 mm 3 μm) was used as the column and the column temperature was set to 30 °C. In the elution gradient, mobile phase A was 0.5% (v/v) of glacial acetic acid prepared in ultrapure water (GFL 2004/Human power 1), and mobile phase B was 99.9% pure methanol. Separation was performed at a flow rate of 0.3 mL/min and an injection volume of 20 µL for 20 min. The gradient started at 0% B, increased to 98% B within 15.5 min, held at 98% B for 0.5 min, and decreased to 0% B for 16 min. Orbitrap HRMS equipped with a heated electrospray ionization interface was used in both positive (Full MS/AIF) and negative (Full MS/AIF) modes.

Stock solutions were prepared by dissolving the compounds in 50% methanol under laboratory conditions and injected into the LC-HRMS system at 10-1000 ppb for the calibration curve. The extracts were filtered through a PTFE syringe filter with a pore size of 0.22 μm and a diameter of 25 mm and 100 µL of the 1.5 mL filtrate was added to 900 µL of a water mixture containing 10% methanol and 0.5% acetic acid. The mixture was homogenized and injected into the device. The compounds in the extracts were identified by comparing retention times and exact masses of the standards.

### Determination of enzyme inhibition

Pancreatic α-amylase (from porcine pancreas, EC 3.2.1.1) inhibition was determined spectrophotometrically using starch as substrate and DNS as reagent. Hydrolysis of p-nitrophenyl-α-D-glucopyranoside, the substrate for α-glucosidase (from *Saccharomyces cerevisiae*, EC 3.2.1.20) inhibition, was determined spectrophotometrically. In enzyme inhibition analysis, buffers were used controls. The amounts of extracts required to inhibit the enzyme by 50% (IC_50_) were calculated and acarbose was used as a positive control (Liu et al. 2013).

### Determination of aroma compounds of olive leaves

The experimental methodology was carried out according to Malheiro et al. (2016) with minor modifications. SPME was used for the extraction of volatiles from lyophilized olive leaves. A homogenized leaf-deionized water mixture (1:1 w/v) and internal standard (4-nananol, 41.46 µg/L) were placed in a 50 mL vial. Extraction was carried out at 50 °C for 45 min. SPME analysis was performed in triplicate. DVB/CAR/PDMS 50/30 µm was used as fiber. The volatile compounds were then analyzed by GC-MS (Shimadzu, QP 2010 ULTRA). Compound separation was carried out using the RXI-5MS capillary column (30 m; 0.25 mm; 0.25 m). The injector was operated in splitless mode at 250 °C, the column temperature was 50 °C for 5 min. These were then adjusted to 270 °C with an increase of 5 °C per minute, to remain constant at this temperature for 5 min. The flow rate of the carrier gas (helium) was 1 mL/min, and the detector and injector temperatures were 250 °C. The library of mass spectroscopy (Wiley 6, NIST 98) and standards were used when diagnosing peaks. Concentrations of volatile compounds were calculated using the formula with the internal standard method (Schneider et al. 2001).

### Statistical analysis

The research was carried out as 2 olive leaves × 3 different solvents × 3 replicates. Duncan’s test from ANOVA tests was applied to significant differences as a result of the analysis of variance for extracts. Results are given as mean±standard deviation.

## Results and discussion

### Total phenol content and antioxidant capacity of olive leaves

**Fig. 1:**
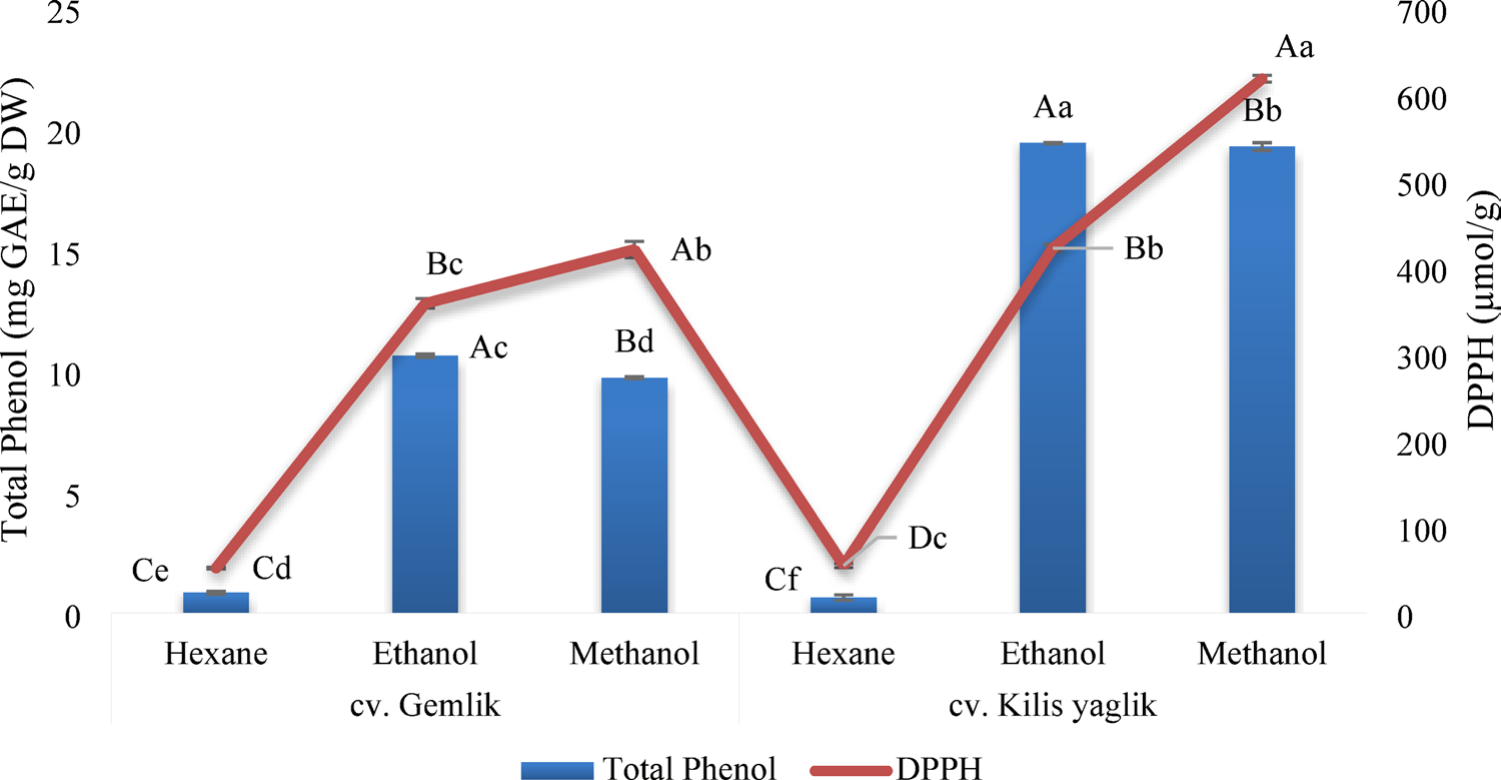
Total phenol and DPPH inhibition of olive leaves. Different capital letters in each variant represent significant differences between solvents; minor letters represent significant differences between extracts (*p* < 0.05)

Depending on the solvent used in the extraction of olive leaves, yields varied and were determined in the range of 1.31–21.33% and 1.41–32.31% in Gemlik and Kilis Yağlık varieties, respectively. In each variety, the lowest extraction yield was obtained with hexane, while the highest yield was obtained with methanol. Erdoğan et al. (2020) reported that the solvent type was effective on the extraction yield of the leaf. The total phenol and antioxidant capacity results of the leaves are given in Fig. 1. In both leaf varieties, the lowest total phenol content was found in hexane extracts, while ethanol and methanol extracts were similar. In parallel with the total phenol content, antioxidant capacity was found to be higher in ethanol extracts. Olive leaves of cv. Kilis Yağlık had approximately 2 times higher total phenol content and showed higher antioxidant capacity than cv. Gemlik. Stupans et al. (2002) reported that oleuropein in olive leaves is a high radical scavenger. Similarly, the high antioxidant capacity of cv. Kilis Yağlık leaves are also related to the amount of oleuropein. In total phenol and DPPH results, the differences between the solvents of the same cultivar and all extracts were statistically significant (*p* < 0.05).

Erdoğan et al. (2020) obtained the highest phenolic content in *n-*hexane, ethyl acetate and methanol extracts of olive leaves with 85.27 mg GAE/g DW in methanol extracts and the lowest with 0.18 µg GAE/g DW in *n-*hexane extract. Iscimen and Hayta (2023) determined the highest total phenolic content of olive leaves obtained from the market as 9.52 mg GAE/mL and DPPH radical scavenging activity as 15.22 mg TE/g. Ronca et al. (2024a, b) reported that the DPPH antioxidant capacity of Picual variety olive leaves collected in Spain varied between 405 and 632 µmol TE/g DW and that this difference depended on sample-solvent ratios and storage times. Kalkan et al. (2023) reported that the DPPH antioxidant capacity of olive leaves was higher in methanol extracts than in ethanol extracts in agreement with the present study. The differences between the results of phenolic substances and antioxidant capacity in the literature are affected by factors such as olive variety, growing conditions of the olive tree, harvest time, storage conditions of the leaf, solvent polarity, solvent concentration, extraction method, extraction conditions, and the analysis method used. Alvares et al. (2024) showed that olive leaves added to olive oil improved the resistance of oils against oxidation, and Zandona et al. (2024) showed that olive leaves added to cheese improved the total phenol content and antioxidant capacity.

### Phenolic compounds of olive leaves by LC-HRMS

Since the total phenol content of hexane extracts with low extraction yields was also low, their phenolic compounds were not determined. Phenolic compounds of ethanol and methanol extracts are given in Table 1. The total phenolic compound content in both olive leaves was determined to be highest in methanol extracts. Similar to the total phenol content of cv. Kilis Yağlık olive leaves, the phenolic compound content is more than 2 times higher than cv. Gemlik. The total amount of phenolic compounds in the extracts was determined as per g DW 247.74 mg in Gemlik ethanol, 258.85 mg in Gemlik methanol, 373.99 mg in Kilis Yağlık ethanol and 441.39 mg in methanol. A total of 27 phenolic compounds, including 18 flavonoids, 8 phenolic acids, and 1 secoiridoid, were determined in olive leaves. The flavonoid, secoiridoid and phenolic acid content of the extracts were determined as 159.15, 85.12 and 3.74 in Gemlik ethanol; 163.61, 90.67 and 4.58 in Gemlik methanol; 231.56, 132.95 and 9.45 in Kilis Yağlık ethanol; 302.83, 136.08 and 2.48 mg/g DW in Kilis Yağlık methanol. Oleuropein, which was determined in the highest amount among the phenolic compounds in the extracts, constituted approximately 35% of the total phenolic compounds. In the literature, oleuropein was reported as the most dominant phenolic compound (Duque-Soto et al. 2022).

**Table 1:** Phenolic compounds of olive leaf extracts identified by LC-HRMS (mg/g DW)

RT*	Phenolic compounds	*R*^2^**	Class	GE	GM	KE	KM
6.33	Protocatechuic acid (3,4-Dihydroxybenzoic acid)	0.9995	Phenolic acid	0.38 ± 0.02	0.28 ± 0.04	ND	ND
6.96	3,4-Dihydroxyphenylacetic acid (DOPAC, Homoprotocatechuic acid)	0.9978	Phenolic acid	1.31 ± 0.13	1.77 ± 0.15	1.42 ± 0.12	ND
7.48	Esculin hydrate	0.9995	Flavonoid	0.05 ± 0.00	0.05 ± 0.00	0.02 ± 0.00	0.03 ± 0.01
7.88	4-Hydroxybenzoic acid	0.9997	Phenolic acid	0.57 ± 0.05	0.50 ± 0.02	ND	ND
8.12	Chlorogenic acid	0.9969	Phenolic acid	ND	ND	0.33 ± 0.21	0.32 ± 0.6
8.62	Vanillic acid	0.9984	Phenolic acid	0.32 ± 0.06	0.26 ± 0.02	ND	ND
9.08	Vicenin 2	0.9996	Flavonoid	1.06 ± 0.25	1.02 ± 0.16	1.15 ± 0.17	1.05 ± 0.11
9.83	Coumaric acid (trans-3-Hydroxycinnamic acid)	0.9995	Phenolic acid	0.41 ± 0.09	0.37 ± 0.05	0.38 ± 0.05	0.41 ± 0.04
10.43	Luteolin 7-rutinoside	0.9985	Flavonoid	2.69 ± 0.21	2.87 ± 0.41	8.73 ± 1.03	9.07 ± 0.95
10.53	Luteoloside (Luteolin 7-glucoside)	0.9961	Flavonoid	33.24 ± 1.17	35.44 ± 0.38	97.20 ± 1.01	126.79 ± 1.11
10.71	Eriodictyol (3,4,5,7-Tetrahydroxyflavanone)	0.9955	Flavonoid	3.99 ± 0.06	4.89 ± 0.05	9.64 ± 0.17	11.47 ± 0.13
10.72	Rutin hydrate M-OH_2_	0.9994	Flavonoid	ND	ND	5.31 ± 0.29	5.48 ± 0.21
10.77	Isoquercitrin (Quercetin 3-glucoside)	0.9951	Flavonoid	0.47 ± 0.07	0.46 ± 0.03	0.43 ± 0.02	0.71 ± 0.09
10.98	Ellagic acid	0.9784	Phenolic acid	ND	ND	3.36 ± 0.03	ND
11.00	Apiin (Apigenin-7-(2-O-apiosylglucoside)	0.9911	Flavonoid	0.11 ± 0.02	0.09 ± 0.01	ND	ND
11.08	Kaempferitrin	0.9975	Flavonoid	2.81 ± 0.98	3.90 ± 0.03	0.88 ± 0.07	1.76 ± 0.16
11.13	Oleuropein	0.9905	Secoiridoid	85.12 ± 1.46	90.66 ± 2.02	132.95 ± 1.60	136.08 ± 1.00
11.17	Apigenin 7-glucoside	0.9916	Flavonoid	1.64 ± 0.20	1.60 ± 0.16	30.79 ± 0.6	29.89 ± 0.53
11.37	Quercetin 3-rutinoside 7-glucoside	0.9964	Flavonoid	48.40 ± 1.25	48.80 ± 1.27	35.38 ± 0.62	72.71 ± 1.03
11.37	Astragalin (Kaempferol 3-glucoside)	0.9964	Flavonoid	48.53 ± 1.02	48.79 ± 1.14	35.41 ± 0.57	39.10 ± 0.21
11.55	Leucoside (Kaempferol 3-sambubioside)	0.9980	Flavonoid	0.34 ± 0.06	0.07 ± 0.01	ND	ND
12.24	Quercetin	0.9972	Flavonoid	2.22 ± 0.12	1.11 ± 0.13	2.76 ± 0.16	0.82 ± 0.16
12.44	Tiliroside	0.9988	Phenolic Acid	0.49 ± 0.05	1.40 ± 0.09	3.97 ± 0.17	1.75 ± 0.12
12.56	Luteolin	0.9984	Flavonoid	10.03 ± 0.28	11.77 ± 0.59	2.94 ± 0.16	3.47 ± 0.25
13.24	Isorhamnetin (Quercetin 3′-methyl ether)	0.9969	Flavonoid	0.93 ± 0.02	0.49 ± 0.09	0.93 ± 0.08	0.45 ± 0.03
13.27	Apigenin (5,7-Dihydroxy-2-(4-hydroxyphenyl)-4 H-chromen-4-one)	0.9971	Flavonoid	0.90 ± 0.02	0.69 ± 0.10	ND	ND
13.38	Diosmetin (Luteolin 4′-methyl ether)	0.9938	Flavonoid	1.73 ± 0.12	1.57 ± 0.21	0.01 ± 0.00	0.03 ± 0.00
	Total			247.74	258.85	373.99	441.39

GE: cv. Gemlik olive leaf ethanol extract, GM: cv. Gemlik olive leaf methanol extract, KE: cv. Kilis Yağlık olive leaf ethanol extract, KM: cv. Kilis Yağlık olive leaf methanol extract, *RT: Retention time, **R^2^: Correlation coefficient, ND: not detected

While protocatechuic acid, 4-hydroxybenzoic acid, vanillic acid, apiin, leucoside, and apigenin were determined only in Gemlik extracts, chlorogenic acid, and rutin hydrate M-OH_2_ were detected only in Kilis Yağlık extracts. Ellagic acid was determined in Kilis Yağlık ethanol extracts.

Ronca et al. (2024a, b) determined oleuropein, quercetin, and luteolin among 10 phenolic compounds in 12 varieties of olive leaves in Portugal. In addition, the same authors reported that the solvent chosen for the extraction of the determined compounds in olive leaves affected the contents of hydroxytyrosol, oleuropein, verbascoside, and luteolin. Magyari-Pavel et al. (2024) reported high amounts of luteolin 7-glucoside as well as oleuropein in 2 different olive leaves originating from Spain and Greece. Vidal et al. (2022) found high amounts of oleuropein, verbascoside, and luteolin-7-O-glucoside and lower amounts of hydroxytyrosol, apigenin-7-O-glucoside, and apigenin in olive leaves. El Adnany et al. (2024) reported that the amounts of 8 different phenolic compounds (hydroxytyrosol, catechin, caffeic acid, vanillin, naringin, oleuropein, quercetin, and kaempferol) identified in olive leaves were affected by thermal processing temperatures and extraction solvent. These changes in phenolic compounds observed in olive leaf extracts are thought to be due to the amount of substance passing into the solvent.

### Enzyme inhibition

Olive leaf extracts (except hexane) showed inhibitory effects on α-glucosidase and α-amylase, which are key enzymes in diabetes (Table 2). For both enzymes, the closest results to acarbose used as a standard were shown by methanol extracts in the cv. Gemlik, while ethanol extracts in the cv. Kilis Yağlık showed results. While the difference between the extracts in enzyme inhibition was statistically significant, the difference between the solvents in Gemlik extracts was insignificant (*p* < 0.05).

**Table 2:** IC_50_ values ​​of α-glucosidase and α-amylase of extracts (mg/kg DW)

	α-glucosidase	α-amylase
GH	5217.19 ± 37.56^a^	7158.75 ± 49.01^a^
GE	193.71 ± 1.17^b^	188.12 ± 2.96^b^
GM	175.63 ± 1.84^b^	92.12 ± 0.57^b^
KH	3457.97 ± 12.32^Aa^	5407.59 ± 15.56^Aa^
KE	147.92 ± 1.77^Bb^	199.03 ± 1.86^Bb^
KM	162.30 ± 0.65^Bb^	244.79 ± 4.06^Bb^
Acarbose	35.74 ± 1.02	40.78 ± 1.13

GH: cv. Gemlik olive leaf hexane extract, GE: cv. Gemlik olive leaf ethanol extract, GM: cv. Gemlik olive leaf methanol extract, KH: cv. Kilis Yağlık olive leaf hexane extract, KE: cv. Kilis Yağlık olive leaf ethanol extract, KM: cv. Kilis Yağlık olive leaf methanol extract, Different capital letters in each variety in the columns represent significant differences between solvents (*p* < 0.05); minor letters represent significant differences between extracts (*p* < 0.05)

Hadrich et al. (2015) reported that hydroxytyrosol and oleuropein in olive leaves showed an inhibition effect against α-glucosidase and α-amylase. Apart from these two main components, luteolin, luteolin-7-O-glucoside, vanillic acid, quercetin, coumaric acid, and apigenin are known to be effective in inhibition (Tadera et al. 2006). In the present study, although only hydroxytyrosol among these phenolic components could not be determined because it was not among the standards, all these components were found to be effective in enzyme inhibition.

Mancak and Koca Caliskan (2024) reported that ethanol extracts of olive leaves showed α-glucosidase inhibition in the range of 89.34–90.54% for 2.5 µg/mL extract and α-amylase inhibition in the range of 49.35–50.11% for 0.625 µg/mL extract. Elayeb et al. (2024) reported that ethanol extract from olive leaf was a potent inhibitor of pancreatic amylase inhibition and found an interesting inhibitory effect on α-amylase with IC_50_ values of 0.75 µg/mL compared to acarbose (IC_50_ = 15.74 µg/mL). Collado-González et al. (2017) determined IC_50_ values of extra virgin olive oils obtained from Arbequina, Picual, Hojiblanca, Cornicabra, and Cuquillo olive varieties between 0.44 and 1.30 µg/mL for α-glucosidase and 1.43–5.85 µg/mL for α-amylase. Considering the enzyme inhibition results in the study and the literature, it was seen that olive leaves can be used as a food supplement in management diabetes.

### Aroma composition of olive leaves

**Fig. 2:**
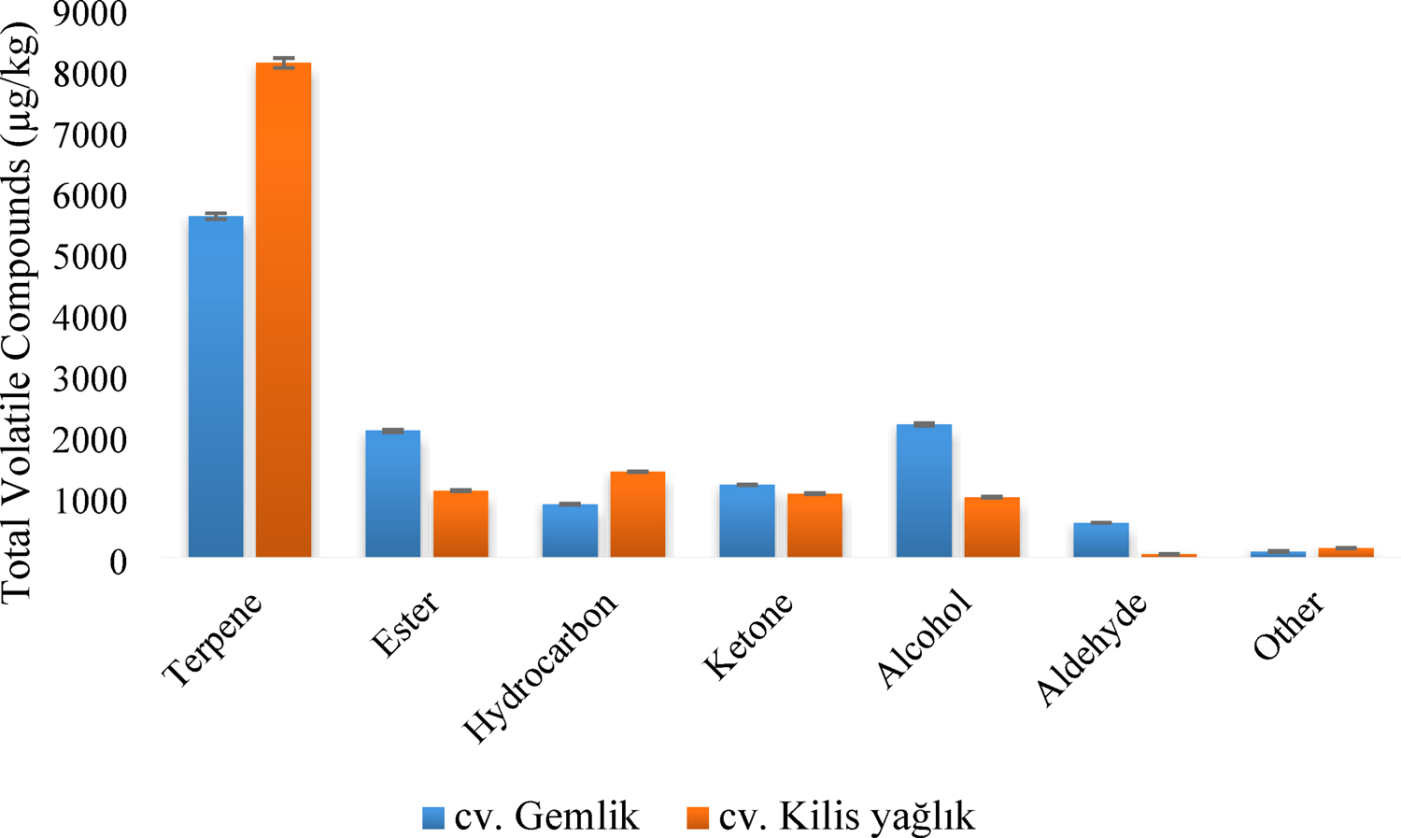
Class distribution of volatile compounds in olive leaves

A total of 52 volatile compounds, including 17 terpenes (monoterpenes and sesquiterpenes), 12 hydrocarbons, 9 alcohols, 5 esters, 4 aldehydes, 3 ketones, and 2 other classes, were detected in lyophilized olive leaves (Table 3). The total amount of volatile compounds was determined as 12 563.01 µg/kg (39 compounds) in the cv. Gemlik and 12 842.04 µg/kg (34 compounds) in the cv. Kilis Yağlık. Terpenes were the volatile compound class detected in the highest amount in the leaves and constituted 44–63% of the total aroma (Fig. 2). Terpenes were followed by alcohols and esters in cv. Gemlik and hydrocarbons and esters in cv. Kilis Yağlık.

**Table 3:** Aroma compounds of olive leaves (µg/kg)

RT*	Aroma Class	Compounds	Gemlik	Kilis Yağlık
1.733	Ester	Acetic acid, ethyl ester	1178.74 ± 15.30	899.20 ± 10.22
1.975	Aldehyde	2-Butenal	103.83 ± 7.40	ND
2.216	Alcohol	1-Penten-3-ol	85.50 ± 4.56	ND
5.421	Aldehyde	(E)-2-Hexenal	177.12 ± 10.25	ND
6.732	Alcohol	4-Heptanol	97.72 ± 8.57	170.97 ± 10.23
10.682	Alcohol	Ethyl-1-hexanol	ND	126.65 ± 11.10
10.692	Aldehyde	(E, E)-2,4-Heptandienal	287.05 ± 15.14	ND
10.762	Hydrocarbon	Decane	ND	151.98 ± 7.52
10.830	Ester	Ethyl hexanoate	140.47 ± 9.73	ND
10.890	Aldehyde	Octanal	ND	50.66 ± 5.36
11.130	Ketone	3-Ethyl-4-Heptanone	158.79 ± 4.12	189.97 ± 8.95
11.792	Terpene	Limonene	494.70 ± 8.96	1076.50 ± 18.62
12.166	Terpene	(Z)-Ocimene	ND	113.98 ± 2.57
13.408	Ketone	4-Nonanone	897.79 ± 10.98	854.87 ± 4.89
14.793	Terpene	Linalool	732.89 ± 8.56	208.97 ± 5.75
15.060	Other	4,8-Dimethyl-3,7-nonadien-2-ol	97.72 ± 5.63	ND
15.306	Alcohol	Phenethyl alcohol	561.88 ± 4.84	ND
16.035	Ketone	Camphor	134.36 ± 4.75	ND
16.194	Hydrocarbon	6-Methyl undecane	250.41 ± 4.62	386.27 ± 10.28
16.330	Alcohol	4-Decanol	ND	50.66 ± 2.56
16.340	Alcohol	2-Methyl-3-hexanol	67.18 ± 8.20	ND
16.771	Ester	Lavandulyl acetate	73.29 ± 5.46	ND
17.031	Other	Tetrahydro-2,2-dimethyl-5-(1-methylpropyl) furan	ND	151.98 ± 14.65
17.109	Alcohol	Terpinen-4-ol	592.42 ± 11.22	nND
17.641	Hydrocarbon	Dodecane	195.44 ± 5.82	265.96 ± 16.84
17.840	Hydrocarbon	(E)-5-Octadecene	ND	246.96 ± 4.25
18.804	Ester	Hexyl-2-methyl butanoate	140.47 ± 9.65	132.98 ± 2.42
19.375	Ester	Linalyl acetate	537.45 ± 8.49	63.32 ± 1.13
20.361	Alcohol	6-Undecanol	354.23 ± 3.65	341.95 ± 1.74
20.515	Hydrocarbon	1,1,2-Trimethyl cycloundecane	79.40 ± 3.94	82.32 ± 5.26
20.656	Hydrocarbon	Tridecane	ND	37.99 ± 3.12
22.205	Alcohol	3,7,11-Trimethyl-1-dodecanol	415.31 ± 5.48	297.62 ± 5.84
22.647	Terpene	Cyclosativene	190.87 ± 4.80	553.46 ± 3.58
22.972	Terpene	α-Copaene	1150.48 ± 16.08	3164.87 ± 39.64
23.282	Terpene	α-Zingiberene	103.83 ± 1.52	ND
24.182	Terpene	β-Caryophyllene	2100.96 ± 35.95	1906.04 ± 16.54
24.396	Terpene	Germacrene	ND	120.32 ± 1.85
24.404	Terpene	β-Copaene	54.97 ± 1.80	ND
24.534	Terpene	(E)-α-Bergamotene	85.50 ± 2.09	ND
24.721	Terpene	β-Sesquiphellandrene	79.40 ± 2.74	ND
24.967	Hydrocarbon	Tritetracontane	ND	94.99 ± 2.49
24.974	Hydrocarbon	2,6,10,15-Tetramethyl heptadecane	146.58 ± 3.99	ND
25.067	Terpene	α-Humulene	428.66 ± 4.52	391.95 ± 8.95
25.643	Terpene	α-Amorphene	ND	31.66 ± 2.95
25.865	Terpene	(Z)-β-Ionone	48.86 ± 4.95	ND
25.933	Terpene	β-Selinene	ND	25.33 ± 0.84
26.105	Hydrocarbon	Tricosane	ND	75.99 ± 2.65
26.111	Hydrocarbon	Tetradecane	48.86 ± 4.56	ND
26.267	Terpene	α-Muurolene	117.18 ± 5.36	448.94 ± 6.05
26.369	Terpene	(E, E)-α-Farnesene	ND	63.32 ± 1.01
28.599	Hydrocarbon	Hexadecane	48.86 ± 2.08	ND
30.001	Hydrocarbon	1-Iodo-dodecane	103.83 ± 2.84	63.32 ± 3.57

*RT: Retention time, ND: not detected

The most abundant aroma compounds identified in olive leaves were β-caryophyllene, acetic acid-ethyl ester, α-copaene, 4-nonanone, and linalool in cv. Gemlik, while α-copaene, β-caryophyllene, limonene, acetic acid-ethyl ester, and 4-nonanone in cv. Kilis Yağlık. The odors given by these dominant compounds are spicy, woody, sweet, fruity, floral, green, and citrus.

Most of the volatile compounds of olive leaves, which are secondary metabolites, are produced via lipoxygenase (Malheiro et al. 2016). In addition, polysaccharide metabolism, fatty acid metabolism, and the conversion of amino acids are also involved in the formation of aroma compounds (Ben-Abdeljelil et al. 2017). Malheiro et al. (2016) identified a total of 39 volatile compounds including 12 esters, 9 sesquiterpenes, 5 alcohols, 4 terpenes, 3 aldehydes, 3 ketones, and 3 hydrocarbons in olive leaves of Cobrançosa, Madural and Verdeal Transmontana varieties at 6 different harvest periods. The total amounts of these compounds ranged from 332 to 6830 µg/100 g depending on harvest time and cultivar. (Z)-3-hexen-1-ol acetate was the most abundant volatile compound in all cultivars. Among terpenes, b-caryophyllene and limonene were reported to be the most important representatives. Ben-Abdeljelil et al. (2017) found that (E)-2-hexenal was the main volatile compound present in fresh olive leaves of the Chemlali cultivar and aldehydes were the most abundant group of volatile compounds in olive leaves. However, Brahmi et al. (2012) did not detect (E)-2-hexenal in olive leaves and reported that alcohols were more abundant than aldehydes. They also reported that (E)-3-hexenol, 3-ethenylpyridine, (E)-β-damassenone, and phenylethyl alcohol compounds were found in high ratios in 3 different varieties of olive leaves and their percentages changed with the drying of the leaves. Similar to the study, linalool, (E, E)-α-farnesene, α-copaene, ocimene, caryophyllene, β-copaene, humulene, germacrene, sesquiphellandrene, and α-muurolene terpenes were also identified in olive leaves by Caselli et al. (2022). It is known that the volatile compounds of olive leaves vary qualitatively and quantitatively according to the extraction method and conditions used (Paskovic et al. 2019), and there is variability in the number of volatile compounds according to the time of extraction (Ben-Abdeljelil et al. 2017).

## Conclusion

The cultivation of Gemlik and Kilis Yağlık olive varieties is common in the south of Turkey. Therefore in this study, ultrasonic-assisted extraction of these olive leaves was carried out using different solvents. The bioactive properties of the extracts and the aroma composition of the leaves were compared. Total phenol and antioxidant capacity were found to be higher in Kilis Yağlık in terms of variety and ethanol extracts in terms of solvent. Among the 27 phenolic compounds determined in olive leaves, including 18 flavonoids, 8 phenolic acids, and 1 secoiridoid, the highest amounts were oleuropein, luteoloside, quercetin 3-rutinoside 7-glucoside and astragalin. Of the volatile compounds belonging to terpene, hydrocarbon, alcohol, ester, aldehyde, ketone, and other classes, 38 (12 563.01 µg/kg) were determined in cv. Gemlik and 33 (12 842.04 µg/kg) in cv. Kilis Yağlık olive leaves. Considering the inhibition of α-amylase and α-glucosidase, it is thought that the use of olive leaves in different forms directly or in enriching foods will contribute to the diets of diabetic patients. Despite the small-scale nature of this study, the extraction approach employed is considered scalable and economically feasible due to the low-cost and continuous availability of olive leaves and the industrial adaptability of ultrasound-assisted extractionThe continuous generation of olive leaves as an agro-industrial by-product ensures their sustainable availability as a raw material for large-scale extract production. It is concluded that olive leaves should be evaluated as a by-product rather than a waste product and more intensive research should be conducted on this subject.

## Data Availability

All data generated or analysed during this study are included in this article.

## Abbreviations

SPME: Solid-phase microextraction
GC-MS: Gas chromatography-mass spectroscopy
GAE: Gallic acid equivalent
DW: Dry weight
DPPH: 1,1-diphenyl-2-picrylhydrazyl
TE: Trolox equivalent
ESI: Electrospray ionization
DNS: Dinitrosalicylic acid
(E): Trans
(Z): Cis

## References

[CR1] Alvares AA, Silva LT, Cavalcante LS, Pires DMA, Machado ICK, Aboy AL, Mello W, Scheid C, Merib J, Garavaglia J (2024) Impact of olive leaves powder addition on extra virgin olive oil: sensory, quality, nutritional and volatile compounds implications. J Am Oil Chem Soc 101:1367–1381. 10.1002/aocs.12841

[CR2] Ben-Abdeljelil Z, Tekaya M, Mechri B, Flamini G, Hammami M (2017) Changes in volatiles of olive tree *Olea europaea* according to season and foliar fertilization. Int J Agric Biol 19:1633–1639. 10.17957/IJAB/15.0496

[CR3] Brahmi F, Flamini G, Issaoui M, Dhibi M, Dabbou S, Mastouri M, Hammami M (2012) Chemical composition and biological activities of volatile fractions from three Tunisian cultivars of olive leaves. Med Chem Res 21:2863–2876. 10.1007/s00044-011-9817-8

[CR4] Caselli A, Favaro R, Petacchi R, Angeli S (2022) Infestation of the gall midge *Dasineura oleae* provides first evidence of induced plant volatiles in olive leaves. Bull Entom Res 112:481–493. 10.1017/S0007485321001000 10.1017/S0007485321001000 34930508

[CR5] Collado-Gonzalez J, Grosso C, Valentao P, Andrade PB, Ferreres F, Durand T, Gil-Izquierdo A (2017) Inhibition of α-glucosidase and α-amylase by Spanish extra virgin olive oils: the involvement of bioactive compounds other than oleuropein and hydroxytyrosol. Food Chem 235:298–307. 10.1016/j.foodchem.2017.04.171 28554640 10.1016/j.foodchem.2017.04.171

[CR6] Duque-Soto C, Quirantes-Piné R, Borrás-Linares I, Segura-Carretero A, Lozano-Sánchez J (2022) Characterization and influence of static in vitro digestion on bioaccessibility of bioactive polyphenols from an olive leaf extract. Foods 11:743. 10.3390/foods11050743 35267376 10.3390/foods11050743 PMC8909904

[CR7] El Adnany EM, Elhadiri N, Mourjane A, Ouhammou M, Hidar N, Jaouad A, Bigaud E, Bitar K, Mahrouz M (2024) Impact of heat treatment and granulometry of olive leaf powder (*Moroccan picholine*) on its phenolic compounds, antioxidant activity and physical–chemical properties. Euro Mediterr J Environ Integr 9:721–732

[CR8] Elayeb R, Bermúdez-Oria A, Lazreg Aref H, Majdoub H, Ritzoulis C, Mannu A, Le Cerf D, Carraro M, Achour S, Fernández Bolaños J, Trigui M (2024) Antioxidant polysaccharide enriched fractions obtained from olive leaves by ultrasound-assisted extraction with α- amylase inhibition, and antiproliferative activities. 3 Biotec 14:92. 10.1007/s13205-024-03939-2 10.1007/s13205-024-03939-2 PMC10899153 38425411

[CR9] Erdoğan S, Küpeli Akkol E, Avcı G (2020) Research on the antioxidant efficiacy of olive (*Olea europaea* L.) leaf using by in vitro methods. Kocatepe Vet J 13:319–326. 10.30607/kvj.698776

[CR10] Hadrich F, Bouallagui Z, Junkyu H, Isoda H, Sayadi S (2015) The α-glucosidase and α-amylase enzyme inhibitory of hydroxytyrosol and oleuropein. J Oleo Sci 264:835–843. 10.5650/jos.ess15026 10.5650/jos.ess15026 26235001

[CR11] Hassan J, Sharifzadeh A, Moghadam S, Shams GH, Aghamohammadi AA, Ghanati K (2024) In-vitro assessment of antifungal and antioxidant activities of olive leaves and fruits at various extraction conditions. Appl Food Biotechnol 11:e10. 10.22037/afb.v10i3.43655

[CR12] Hassen IE, Casabianca H, Hosni K (2015) Biological activities of the natural antioxidant oleuropein: exceeding the expectation – a mini-review. J Funct Food 18:926–940. 10.1016/j.jff.2014.09.001

[CR13] Iscimen EM, Hayta M (2023) Zeytin yapraklarından fenolik bileşenlerin mikrodalga destekli ekstraksiyonu ve kinetiği ile ekstraktların antioksidan özellikleri. Akademik Gıda 21:233–242. 10.24323/akademik-gida.1382919

[CR14] Kalkan M, Aygan A, Comlekcioglu N, Comlekcioglu U (2023) Investigation of some bioactive properties and antimicrobial activity of *Olea europaea* leaves. TURJAF 11:496–504. 10.24925/turjaf.v11i3.496-504.5828

[CR15] Liu S, Li D, Huang B, Chen Y, Lu X, Wang Y (2013) Inhibition of pancreatic lipase, α-glucosidase, α-amylase, and hypolipidemic effects of the total flavonoids from *Nelumbo nucifera* leaves. J Ethnopharmacol 149:263–269. 10.1016/j.jep.2013.06.034 23811214 10.1016/j.jep.2013.06.034

[CR16] Magyari-Pavel IZ, Moacă EA, Avram S, Diaconeasa Z, Haidu D, Stefănut MN, Rostas AM, Muntean D, Bora L, Badescu B, Iuhas C, Dehelean CA, Danciu C (2024) Antioxidant extracts from Greek and Spanish olive leaves: antimicrobial, anticancer and antiangiogenic effects. Antioxidants 13:774. 10.3390/antiox13070774 39061845 10.3390/antiox13070774 PMC11273738

[CR17] Malheiro R, Casal S, Cunha SC, Baptista P, Pereira JA (2016) Identification of leaf volatiles from olive (*Olea europaea*) and their possible role in the ovipositional preferences of olive fly, *Bactrocera oleae* (Rossi) (Diptera: Tephritidae). Phytochemistry 121:11–19 26603276 10.1016/j.phytochem.2015.10.005

[CR18] Mancak M, Koca Çalışkan U (2024) What do people prefer to support diabetes treatment in Turkiye? A study on olive leaf and diabetes. J Fac Pharm Ankara Univ 48:436–455. 10.33483/jfpau.1378992

[CR19] Pasković I, Soldo B, Talhaoui N, Palčić I, Brkljača M, Koprivnjak O, Majetić Germek V, Ban D, Klanjac J, Franić M, Žurga P, Grozić K, Lukić I, Goreta Ban S (2019) Boron foliar application enhances oleuropein level and modulates volatile compound composition in olive leaves. Sci Hortic 257:108688. 10.1016/j.scienta.2019.108688

[CR20] Ronca CL, Duque-Soto C, Samaniego-Sánchez C, Morales-Hernández ME, Olalla-Herrera M, Lozano-Sánchez J, Giménez Martínez R (2024a) Exploring the nutritional and bioactive potential of olive leaf residues: a focus on minerals and polyphenols in the context of Spain’s olive oil production. Foods 13:1036. 10.3390/foods13071036 38611342 10.3390/foods13071036 PMC11012209

[CR21] Ronca CL, Marques SS, Ritieni A, Gimenez-Martinez R, Barreiros L, Segundo MA (2024b) Olive oil waste as a source of functional food ingredients: assessing polyphenolic content and antioxidant activity in olive leaves. Foods 13:189. 10.3390/foods13020189 38254490 10.3390/foods13020189 PMC10814828

[CR22] Salik MA, Cakmakci S (2021) Zeytin (*Olea europaea* L.) yaprağının fonksiyonel özellikleri ve gıdalarda kullanım potansiyeli. Gıda 16:1481–1493. 10.15237/gida.GD21133

[CR23] Schneider R, Rauzungles A, Augier C, Baumes R (2001) Monoterpenic and norisoprenoid glycoconjugates of *Vitis vinifera* L. cv. Melon B. as precursors of odorants in Muscadet wines. J Chromatogr A 936:145–157. 10.1016/S0021-9673(01)01150-5 11760996 10.1016/s0021-9673(01)01150-5

[CR24] Singleton VL, Orthofer R, Lamuela-Raventos RM (1999) Analysis of total phenols and other oxidation substrates and antioxidants by means of folin-ciocalteu reagent. Methods Enzymol 299:152–178. 10.1016/S0076-6879(99)99017-1

[CR25] Stupans I, Kirlich A, Tuck KL, Hayall PJ (2002) Comparison of radical scavenging effect, inhibition of microsomal oxygen free radical generation, and serum lipoprotein oxidation of several natural antioxidants. J Agric Food Chem 50:2464–2469. 10.1021/jf0112320 11929315 10.1021/jf0112320

[CR26] Tadera K, Minami Y, Takamatsu K, Matsuoka T (2006) Inhibition of α-glucosidase and α- amylase by flavonoids. J Nutr Sci Vitaminol 52:149–153. 10.3177/jnsv.52.149 16802696 10.3177/jnsv.52.149

[CR27] Thaipong K, Boonprakob U, Crosby K, Cisneros-Zevallos L, Byrnec L (2006) Comparison of ABTS, DPPH, FRAP, and ORAC assays for estimating antioxidant activity from guava fruit extracts. J Food Compos 19:669–675. 10.1016/j.jfca.2006.01.003

[CR28] Vidal AM, Moya M, Alcalá S, Romero I, Espínola F (2022) Enrichment of refined olive oils with phenolic extracts of olive leaf and exhausted olive pomace. Antioxidants 11:204. 10.3390/antiox11020204 35204087 10.3390/antiox11020204 PMC8868085

[CR29] Zandona E, Vrankovic L, Pedisic S, Vukušic Pavicic T, Dobrincic A, Marušic Radovcic N, Lisak Jakopovic K, Blažic M, Barukcic Jurina I (2024) Production of acid and rennet coagulated cheese enriched by olive (*Olea europaea* L.) leaf extract determining the optimal point of supplementation and ıts effects on curd characteristics. Foods 13:616. 10.3390/foods13040616 38397592 10.3390/foods13040616 PMC10887763

[CR30] Zhang CC, Xin XT, Zhang JM, Zhu SL, Niu EL, Zhou ZJ, Liu DQ (2022) Comparative evaluation of the phytochemical profiles and antioxidant potentials of olive leaves from 32 cultivars grown in China. Molecules 27:1292. 10.3390/molecules27041292 35209081 10.3390/molecules27041292 PMC8878581

